# Distal to proximal: a continuum of drivers shaping tree growth and carbon partitioning

**DOI:** 10.1111/nph.70516

**Published:** 2025-09-04

**Authors:** Antoine Cabon

**Affiliations:** ^1^ Swiss Federal Research Institute WSL Zürcherstrasse 111 8903 Birmensdorf Switzerland; ^2^ Estación Experimental del Zaidín – Consejo Superior de Investigaciónes Cientificas Calle Profesor Albareda 1 Granada 18008 Spain

**Keywords:** allocation, carbon partitioning, photosynthesis, source vs sink limitation, trait coordination, transport‐resistance, tree growth, turgor

## Abstract

The relationship between tree carbon (C) assimilation and growth is central to understanding tree functioning and forecasting forest C sequestration, yet remains unresolved. The long‐standing debate over C *source* vs *sink* limits to growth has yielded invaluable insight, but rests on a false dichotomy. Reframing this issue in terms of *distal*‐*to*‐*proximal* processes driving sink activity and placing it within a broader understanding of C partitioning offers new insights. Building on transport‐resistance theory, I outline a framework where plant resource economies shape spatial gradients of resource availability along the leaf‐to‐root axis, thereby regulating local sink activity. This spatially explicit, trait‐informed perspective aligns with optimality theory and provides a mechanistic link between C partitioning and the plant functional trait spectrum. By moving beyond binary limitations and emphasizing integrated physiological processes, this approach can improve understanding of tree function and biomass increment under climate change.

**Table:** 

	Contents	
	Summary	729
I.	Distal to proximal drivers of sink activity	729
II.	Carbon partitioning matters – twice	730
III.	A mechanistic framework for optimal carbon partitioning	730
IV.	Integrated processes of carbon partitioning	732
V.	Coordinated and dynamic adjustment to environment	733
VI.	Conclusion and outlook	733
	Acknowledgements	734
	References	734

## I. Distal to proximal drivers of sink activity

Forests play a central role in the global carbon (C) cycle, yet the future size of the forest C pool remains a major uncertainty in projections of forest dynamics and climate change. A key aspect of this uncertainty concerns the fate of assimilated C (Pugh *et al*., 2020). While a strong link between photosynthetic C assimilation by photosynthesis and sequestration in tissue growth has long been assumed, Körner and colleagues proposed that these two processes may be largely decoupled (Fatichi *et al*., 2014; Körner, 2015). Instead, growth could be controlled by factors such as turgor, temperature, or stoichiometric constraints on cell expansion and division. This debate – commonly framed as *source* vs *sink* limitation to growth – remains unresolved (Cabon *et al*., 2022; Gessler & Zweifel, 2024; Trugman & Anderegg, 2025).

Many Earth system models notably adopt a source perspective, leading to strong coupling between simulated photosynthesis and growth. This results in projections of substantial forest C sequestration, primarily due to the CO_2_ fertilization effect (Friend *et al*., 2019). Yet evidence for sustained tree growth increases under elevated CO_2_ is mixed (Walker *et al*., 2021). Under the alternative assumption that growth is controlled by factors other than photosynthesis, the CO_2_ fertilization effect on biomass could be modest, with surplus C redirected to shorter‐lived sinks contributing less to long‐term sequestration (Körner, 2022).

**Fig. 1:**
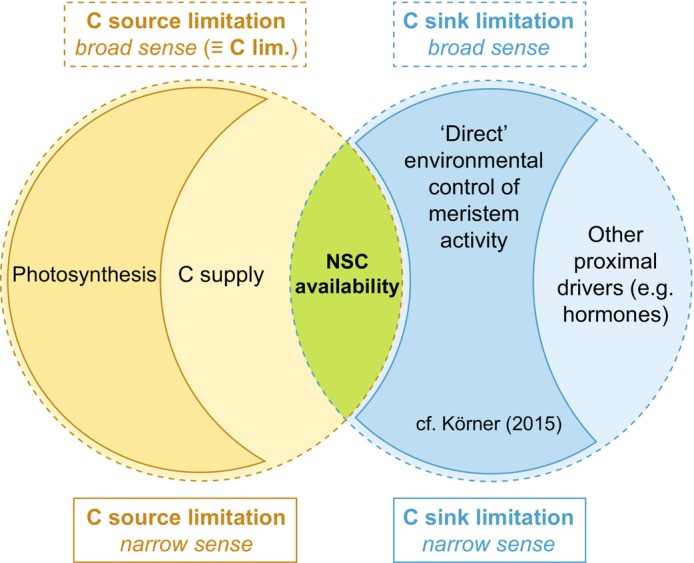
Definitions of carbon source and sink limitations to tree growth are overlapping and inconsistent. In a narrow sense (solid orange), source limitation refers to control by photosynthesis; in a broader sense (dashed orange), it includes the overall supply (including that from storage) and resulting local availability of nonstructural carbohydrates (NSCs). Sink limitation is often narrowly defined (solid blue) as direct environmental control of meristem activity; yet interpreted literally, sink limitations more broadly (dashed blue) include all proximate drivers of growth, such as sink turgor pressure, but also sink NSC availability.

Part of the debate nevertheless arises from inconsistent interpretations of C source and sink limitations (Fig. 1). For example, Cabon *et al*. (2022) define the source *narrowly* as photosynthesis, so decoupling between annual photosynthesis and radial growth implies growth, for example, supported by storage remobilization, is not always source‐limited at that timescale. Others adopt a *broader* view, extending the definition of C source to storage remobilization, whereby source limitation is synonymous with overall C limitation (Gessler & Zweifel, 2024; Trugman & Anderegg, 2025).

Sink limitation, on the other hand, is often *narrowly* defined as direct environmental control of meristem activity – for example, constraints imposed by nutrients, turgor, or temperature (Körner, 2015). However, C and nutrient availability or turgor pressure at the sink all similarly depend on uptake by source organs. Interpreted literally, sink limitation thus *broadly* encompasses all proximate controls of tissue formation, including C availability. Albeit subtle, these differences in perspective can lead to divergent conclusions regarding the importance of source vs sink limitations and complicate hypothesis testing and community consensus.

I suggest reframing the source–sink framework in terms of distal and proximal controls on sink activity. In that view, growth is necessarily sink‐limited in a proximate sense, and the question instead becomes: what distal and proximal processes drive sink activity and biomass increment at scales that matter for the forest C cycle? Importantly, most empirical evidence relates to radial growth at breast height, a key biomass component, but not the sole or even a dominant sink. While total sink activity (including storage and exports) must ultimately match C assimilation (Trugman & Anderegg, 2025), individual sinks, which contribute variably to long‐term biomass, may be regulated by distinct drivers. Understanding these drivers is essential for clarifying how C is partitioned among sinks, which in turn drives tree function and C turnover.

## II. Carbon partitioning matters – twice

**Fig. 2:**
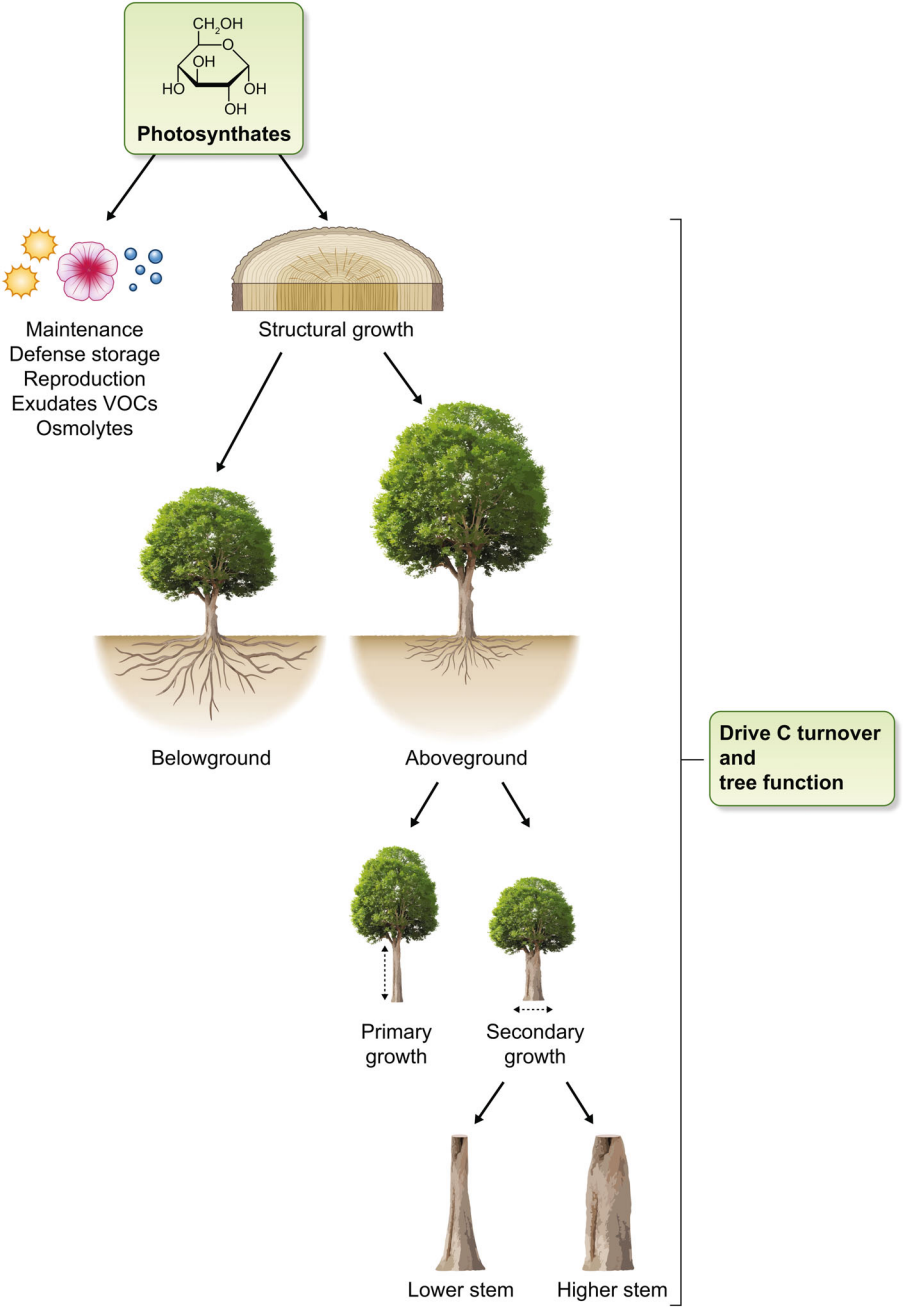
Photosynthate partitioning across functions and scales is shaped by ontogeny and environment, influencing tree function and C turnover. Despite a certain coordination, each partitioning step is plastic. As a result, the activity of a single sink, like stem radial growth at breast height, can be substantially decoupled from photosynthesis.

Woody biomass stores the largest share of live C in forests, yet only a small fraction of photosynthates is partitioned to radial growth (Litton *et al*., 2007; Kannenberg *et al*., 2024). Trees must allocate C across essential functions – growth, reproduction, defense, and storage – and between compartments across scales, from organs to tissues – for example, below vs aboveground, primary vs secondary growth (Fig. 2). Subtle shifts in partitioning can thus decouple photosynthesis from specific sinks like radial stem growth (Cabon *et al*., 2022). Because stem wood is long‐lived, allocation away from it reduces C residence time and tree biomass accumulation (Kannenberg *et al*., 2022). In addition, partitioning shapes forest biomass indirectly, by influencing tree function and survival (Gessler & Zweifel, 2024).

Carbon partitioning across functions responds to environmental conditions – such as drought, temperature, and competition – and ontogeny (Litton *et al*., 2007; Kannenberg *et al*., 2025). Under stress, trees commonly allocate resources toward reproduction, defense, and storage at the expense of structural growth (Blumstein *et al*., 2022; Monson *et al*., 2022; Vincent & Ibáñez, 2024). Future climate may therefore drive long‐term shifts in partitioning patterns, potentially compromising radial growth (Hacket‐Pain *et al*., 2025).

Partitioning between compartments also varies with climate and plant development, as reflected in metrics like above‐to‐belowground biomass (Ma *et al*., 2021), height‐to‐diameter (Tumber‐Dávila *et al*., 2022) or sapwood‐to‐leaf area ratios (Mencuccini *et al*., 2019). These patterns align with plant trait spectra that define ecological strategy and stress tolerance (Rowland *et al*., 2023). For example, adaptation or acclimation to drought often involves greater root investment, slower growth, and production of denser, less conductive tissues (Mencuccini *et al*., 2019; Rowland *et al*., 2023). Thus, partitioning is coordinated with other traits to maintain function and resource use efficiency under stress (Rosana *et al*., 2021; Monson *et al*., 2022).

## III. A mechanistic framework for optimal carbon partitioning

Because partitioning directly influences fitness, it is expected to tend toward optimality under natural selection (Franklin *et al*., 2020). The functional equilibrium hypothesis thus proposes that plants allocate more to organs acquiring the most limiting resource – light, water, or nutrients – enabling optimized fitness (Bloom *et al*., 1985; Franklin *et al*., 2020). Though simple, this principle aligns with observations and models that optimize fitness proxies (Mencuccini *et al*., 2019; Potkay *et al*., 2021). More generally, optimality approaches suggest that plants adjust allocation dynamically to maximize fitness under prevailing conditions, which helps explain strategies like favoring storage over growth during stress (Stefaniak *et al*., 2024).

Optimality approaches therefore provide insight into the eco‐evolutionary logic of C partitioning and support predictive models without detailed process representation (Franklin *et al*., 2020). However, their reliability depends on the choice of fitness proxy, which is uncertain and may not be valid under novel conditions. Here, mechanistic models can constrain and complement optimality approaches, yet remain scarce (Fatichi *et al*., 2019; Merganičová *et al*., 2019).

Transport‐resistance models, initiated by Thornley (1972), remain among the most promising attempts to mechanistically represent whole plant partitioning. These models simulate internal transport of C, water, and nutrients, with transport‐resistance shaping resource availability along the network. Local sink strength and consumption modulate fluxes, leading to emergent equilibria in partitioning. Furthermore, because fluxes depend on both uptake and transport, the equilibrium reflects environmental context and internal architecture (Thornley, 1972; Dewar, 1993).

Despite their promise, TR models remain mostly conceptual due to historical difficulty in measuring transport properties and physiological parameters, holding back their use in vegetation modeling (Merganičová *et al*., 2019). However, advances in understanding and modeling of coupled water and carbon economies and proximal growth drivers (e.g. Hölttä *et al*., 2010; Potkay *et al*., 2022) provide the tools to refine and extend this approach. Representing source–sink coupling and transport allows for dynamic modeling of C partitioning and coordinated development along the leaf‐to‐root axis. The next sections illustrate this approach under varying aridity and atmospheric CO_2_. For clarity, I focus on C and water, but nutrients and temperature can be integrated (Dewar, 1993; Parent & Tardieu, 2012).

## IV. Integrated processes of carbon partitioning

The exchange of water for C via stomata is a fundamental trade‐off in plant function. Photosynthesis and transpiration generate opposing gradients along the leaf‐to‐root axis (Hölttä *et al*., 2006). Resistance to water flow in the xylem is associated with increasingly negative water potential toward the leaves. Because osmotic adjustment is limited along this axis, declining water potential leads to reduced turgor pressure from the root to the leaf (Woodruff *et al*., 2004; Woodruff & Meinzer, 2011). As turgor drives cell expansion, it is closely linked to cell division and tissue growth (Cabon *et al*., 2020b; Peters *et al*., 2021). Thus, the drop in axial turgor pressure contributes to height‐related declines in organ size, tissue density, and shoot growth (Meinzer *et al*., 2008; Woodruff & Meinzer, 2011).

In the phloem, the buildup of nonstructural carbohydrates (NSCs) in the leaves drives the flow and gradient of recently assimilated C to downward sinks (Hölttä *et al*., 2006). Although trees maintain a large total NSC pool, sink activity relies primarily on a fast‐turnover pool composed of recent C, while a substantial portion of NSCs is stored long term (Dietze *et al*., 2013; Hart *et al*., 2024). The remobilization of these older reserves can occur during prolonged periods of low photosynthesis or recovery, but is often associated with reduced vigor (Carbone *et al*., 2013; Peltier *et al*., 2023). The gradient of fresh C supply from leaf to root thus induces greater use of old C and slower recovery of NSC stores belowground than aboveground (Landhäusser & Lieffers, 2012; Hilman *et al*., 2024). Under typical conditions, fresh C supply likely drives a leaf‐to‐root gradient in available C, though the distribution of C stores may contribute more largely during periods of low assimilation.

**Fig. 3:**
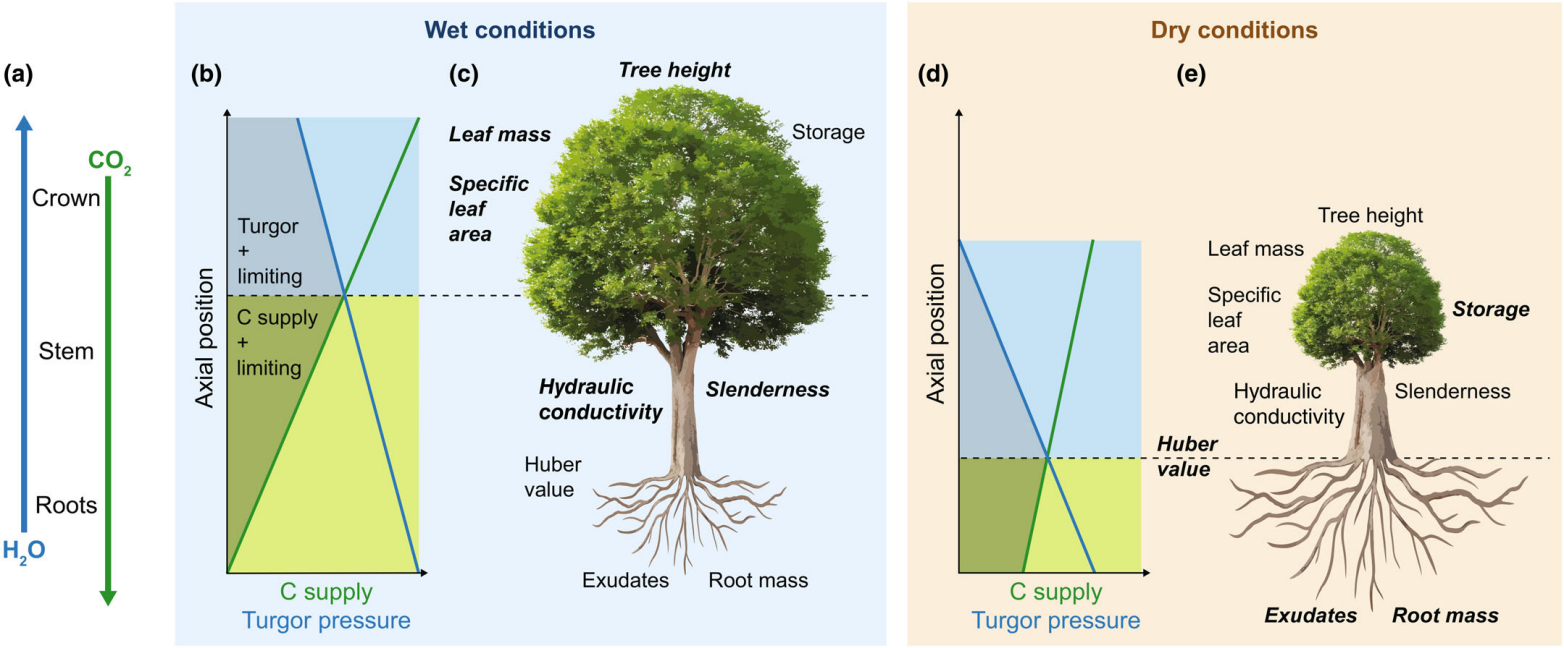
Resource (here C and water) uptake, transport, and consumption shape resource distribution along the leaf‐to‐root axis, influencing C partitioning and plant functional traits under changing environmental conditions. (a) Photosynthesis and transpiration generate C and water fluxes through the leaf‐to‐root continuum. (b, d) These produce opposing gradients of fresh C supply and turgor pressure (green and blue lines), which modulate local sink activity. The transition between C and turgor limitation (dashed line) and the resulting C partitioning profile (shaded area) shift depending on environmental context (here wet vs dry). (c, e) These dynamics influence C partitioning and associated traits. Italicized and bold traits indicate which condition (wet or dry) leads to a higher trait value.

Together, these opposing gradients in turgor pressure and fresh C supply result in a shift in the dominant limitation on growth along the plant axis (Fig. 3), analogous to temporal transitions during organ development (Pantin *et al*., 2011). This axial shift reflects a dynamic equilibrium of biomass partitioning: increased allocation to the crown not only enhances photosynthesis but also raises transpiration, lowering turgor pressure aboveground and pushing the turgor–C transition downward. By contrast, enhanced turgor limitation in the canopy favors belowground partitioning, which improves water (and nutrient) uptake but heightens C limitation belowground, shifting the transition point upward.

In this framework, internal resource gradients act as both integrative signals and active drivers of biomass partitioning. Their interaction forms stabilizing feedback that coordinates whole‐plant responses under changing environmental conditions. Although partly reliant on passive mechanisms, these gradients are tightly regulated through uptake and transport. For example, stomatal regulation may serve to optimize turgor‐driven growth rather than solely maximizing assimilation (Potkay & Feng, 2023). While the current framework suggests a central role of resource control of sink activity in coordinating whole plant C partitioning and trait plasticity, it is complementary to other known mechanisms not implicitly considered here (e.g. Tardieu *et al*., 2011). Notably, hormones, beyond being key controls of plant development, could interact with the transport‐resistance framework by tuning sink strength, modulation of stomatal conductance, or phloem unloading and coordination of source and sink activities (Tardieu *et al*., 2010; Yu *et al*., 2015).

## V. Coordinated and dynamic adjustment to environment

By altering resource uptake, environmental conditions shape axial resource gradients and thus steer the equilibrium partitioning (Fig. 3). Aridity, a major driver of plant allocation patterns, reduces soil water availability while increasing atmospheric demand. This steepens the water potential gradient and exacerbates turgor limitation in the crown (Mencuccini *et al*., 2024). As shoot and leaf growth are more constrained (Meinzer *et al*., 2008; Woodruff & Meinzer, 2011), surplus carbon is reallocated to sinks further downstream (Prescott *et al*., 2020), promoting lower stem and belowground investment (Fig. 3a vs b). This is consistent with widespread increases in root‐to‐shoot or sapwood‐to‐leaf ratios in arid conditions (Mencuccini *et al*., 2019; Tumber‐Dávila *et al*., 2022). Although osmotic adjustment could potentially buffer declining turgor pressure – especially under large C availability – evidence suggests limited plasticity and role in sustaining growth (Bartlett *et al*., 2014; Cabon *et al*., 2020b). Instead, high meristem sensitivity to water status may have been selected through evolution as a mechanism to prioritize belowground or storage allocation during drought, thereby improving fitness (Stefaniak *et al*., 2024).

The effect of elevated CO_2_ on growth and partitioning is more variable, depending on interactions with other limiting factors (Fatichi *et al*., 2016). Elevated CO_2_ enhances photosynthesis, which, in the present framework, preferentially benefits C‐limited sinks. In wet (and nutrient‐rich) conditions, limited turgor constraints enable most sinks on the leaf‐to‐root axis to benefit from increased C supply. However, because of stronger C limitation, downstream sinks are likely to be more sensitive (Minchin & Lacointe, 2005). In this scenario, biomass increases aboveground but potentially more so belowground. Alternatively, in dry conditions, widespread turgor limitation restricts the benefit of increased C supply to belowground sinks like roots, or less turgor‐sensitive sinks like storage or exudates (Prescott *et al*., 2020). This may explain the lack of stem growth response but increased belowground allocation under elevated CO_2_ in dry conditions (Wang *et al*., 2022). Reduced stomatal conductance and evapotranspiration under elevated CO_2_ can nevertheless partially ameliorate plant water status, especially in dry conditions (Wang *et al*., 2022). Accordingly, turgor pressure increase toward the leaves may lead to enhanced primary growth, which aligns with observed CO_2_‐driven vegetation greening, particularly pronounced in arid landscapes (Zhu *et al*., 2016).

Partitioning is tightly coordinated with other functional traits. Within this framework, two main pathways govern this coordination. First, the controls on sink activity directly shape tissue structure. For example, during drought, reduced turgor and elevated C availability not only shift allocation belowground but also favor denser tissue formation (Meinzer *et al*., 2008; Cartenì *et al*., 2018; Cabon *et al*., 2020a). Denser tissues, commonly observed in dry conditions in association with a high water supply‐to‐demand tissue ratio, reduce hydraulic conductance but potentially enhance resistance to cavitation, helping to sustain more negative water potentials (Mencuccini *et al*., 2019; Xu *et al*., 2021). Second, traits governing C and water economies feed back on internal gradients. Hence, an acquisitive trait syndrome characterized by low per‐leaf‐area photosynthetic capacity and high hydraulic conductance should be associated with low (per‐leaf‐area) carbon supply but high turgor pressure. This pattern favors aboveground allocation and supports large leaf areas with relatively low conductive tissue investment (Mencuccini *et al*., 2019; Xu *et al*., 2021).

## VI. Conclusion and outlook

Reframing source–sink limitation as a continuum of distal‐to‐proximal drivers of sink activity offers new insight into the specific bottlenecks between carbon assimilation and allocation to individual sinks. Understanding where and when these bottlenecks arise is a research priority. Building on transport‐resistance theory and incorporating refined physiological knowledge provides a promising path forward. In this framework, leaf‐to‐root resource gradients are tightly regulated as a part of the plant resource economy and enable whole plant control of sink activity. Emerging patterns suggest broad agreement with plant optimality and functional trait theories. Notably, this framework mechanistically captures two key aspects of C partitioning: (1) partitioning dynamically tracks environmental conditions, tending toward functional optimality; (2) partitioning is coordinated across the whole plant in conjunction with trait syndromes.

Testing this framework will require a combination of *in situ* observations, controlled experiments, and process‐based modeling. Time series of sink activity at different positions along the axis (e.g. radial growth) are essential for evaluating whether different drivers dominate. Such observations are particularly valuable in Free‐Air CO_2_ Enrichment experiments and eddy‐covariance sites, where the link with photosynthesis can be assessed. The framework notably predicts a stronger association between photosynthesis and radial growth should be strongest in the roots, whereas upper stem and branch growth should correlate more closely with soil water availability and atmospheric demand or nutrient supply. Additionally, as reliance on stored carbon increases, the age of C used for growth is expected to increase from leaves to roots. The steepness of this gradient should scale with the intensity of non‐C limitations (e.g. Hilman *et al*., 2024). Concomitant estimates of photosynthesis, NSC concentration, sap flow, soil and leaf water potential (among others), together with observations of sink activity across multiple temporal scales, are essential for validation of trait‐based models of C partitioning. Once validated locally, these models could be embedded into global vegetation models to validate and forecast whole‐tree C partitioning across broader spatial and climatic gradients.

## Competing interests

None declared.

## Disclaimer

The New Phytologist Foundation remains neutral with regard to jurisdictional claims in maps and in any institutional affiliations.
